# Unlocking the Bioactive Potential of *Streptomyces*: A Multidisciplinary Approach to Strain Improvement

**DOI:** 10.1002/mbo3.70379

**Published:** 2026-08-24

**Authors:** Anum Goraya, Anila Fariq, Aneela Fatima, Syed Hussain Imam Abidi, Sohail Ameer Marwat, Gholamreza Abdi

**Affiliations:** ^1^ PCSIR Laboratories, Islamabad Pakistan Council of Scientific and Industrial Research Islamabad Pakistan; ^2^ Department of Biotechnology, Persian Gulf Research Institute Persian Gulf University Bushehr Iran

**Keywords:** actinomycetes, adaptive laboratory evolution, genetic engineering, secondary metabolism, *Streptomyces*

## Abstract

*Streptomyces* is a genus of Gram‐positive bacteria renowned for its remarkable ability to produce a diverse array of secondary metabolites (SMs), many of which possess significant pharmaceutical and agricultural value. Unlocking the full biopotential of *Streptomyces* is increasingly important in the quest for new therapeutics and sustainable solutions to global challenges, such as antibiotic resistance and food security. This review paper aims to bridge gaps in the literature by providing a comprehensive, integrative overview of recent advances in *Streptomyces* strain improvement. It discusses optimization of SM production in *Streptomyces* through a multidisciplinary approach. It also highlights the integration of traditional methods with modern genetic and metabolic engineering techniques to improve biosynthetic pathways, as well as adaptive laboratory evolution for strain development, systems, and fermentation, aiming to maximize the production of valuable bioactive compounds. While previous studies have explored individual aspects of strain optimization, this review presents a unified perspective on how these strategies can be applied effectively. This review not only enhances the understanding of *Streptomyces* biopotential but also addresses the need for a holistic framework that considers both established and emerging techniques in strain improvement. By filling these gaps, this review contributes to a more complete understanding of how to optimize *Streptomyces* for the production of valuable bioactive compounds, guiding future innovations and applications in biotechnology and pharmaceuticals.

## Graphical abstract

This paper aims to address critical gaps in the literature by providing a comprehensive, integrative synthesis of recent advances in *Streptomyces* strain improvement, with a particular focus on optimizing secondary metabolite production through a multidisciplinary approach.

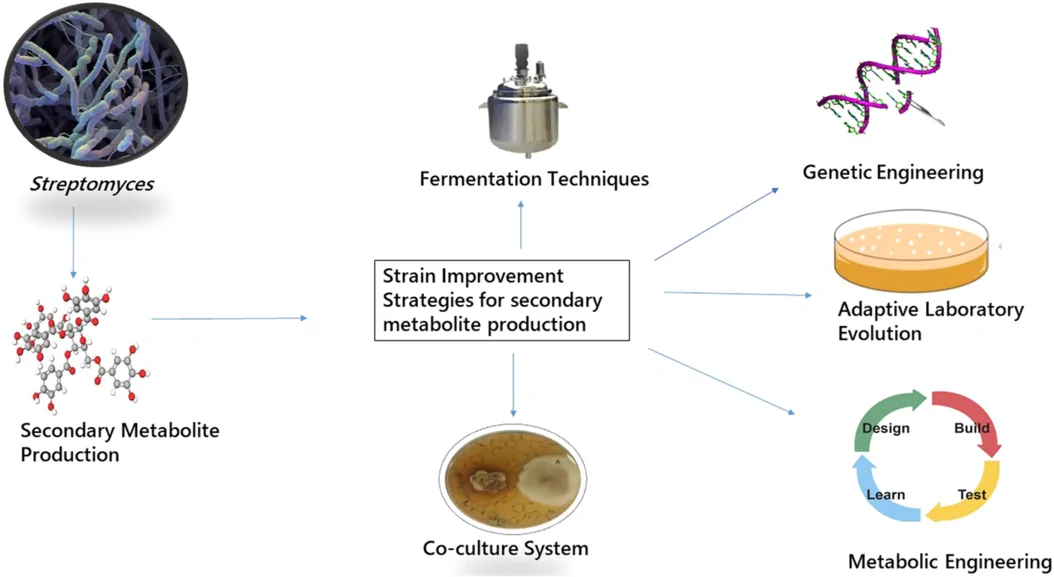

## 1 Introduction

Microorganisms are natural producers of bioactive compounds, known as secondary metabolites (SMs). These metabolites play a substantial role in medicine, agriculture, and stock breeding as they exhibit antibacterial, antiparasitic, and anticancerous activities (Hutchings et al. 2019). Among all microorganisms, actinomycetes are predominant in the synthesis of bioactive compounds. Statistical data indicate that of the 20,000–30,000 microbial SMs, more than 10,000 originate from actinomycetes (Dimri et al. 2020). *Streptomyces*, a family of actinomycetes, is a group of Gram‐positive, filamentous soil saprophytes with an exceptionally high GC content of 70% or higher (Hopwood 2006). Currently, StreptomeDB 4.0 contains over 8500 natural products (NPs) originating from ∼3900 *Streptomycetes* (Y. Feng et al. 2025; Lucas et al. 2012). Nearly 70% of existing clinical antibiotics, including vancomycin, daptomycin, and tetracycline, as well as antifungals (Law et al. 2019), antiparasitics (such as avermectins), anticancer drugs (like doxorubicin), immunosuppressants, and other compounds, have their origins in *Streptomyces* (Chater 2016). Each of these metabolite categories is crucial to different industries and requires large‐scale production to meet the demands of an expanding market. Therefore, it has become indispensable to engineer the *Streptomyces* species for the bulk production of commercially important industrial metabolites. Consequently, over the past few decades, the biosynthetic pathways of *Streptomyces* have been a key focus of strain improvement, metabolic engineering, and bioengineering (Y. Chen et al. 2020).

Research on the *Streptomyces* genome has shown the presence of large linear chromosomes and distinct linear plasmids (Omura et al. 1986; Nikolaidis et al. 2023; Mohite et al. 2025). Whole‐genome sequencing and genome‐mining studies have demonstrated that *Streptomyces* genomes contain a large number of biosynthetic gene clusters (BGCs), many of which remain transcriptionally silent or poorly expressed under laboratory conditions (Aigle et al. 2014; Bachmann et al. 2014; Baltz 2008; Challis 2014; Helfrich et al. 2014; Milshteyn et al. 2014; Olano et al. 2014; Owen et al. 2015; Belknap et al. 2020; Navarro‐Muñoz et al. 2020). Typically, every SM of *Streptomyces* is encoded by extensive BGCs ranging from 20 to 30 genes (Bentley et al. 2002; Ikeda et al. 2003; Oliynyk et al. 2007; N. Lee et al. 2021; Y. Lee, Hwang, et al. 2024), which can be as large as 100 kb (August et al. 1998; Donadio et al. 2002).

Only a small portion of the Earth's surface has been sampled for isolating actinomycetes (Baltz 2005). Various Metagenomic studies have shown that novel polyketide (PK) and nonribosomal peptide pathways for SM production remain underexplored (Charlop‐Powers et al. 2015; N. Lee et al. 2020). Mainly, gene clusters are the primary focus for enhancing the production of SMs (Bekker et al. 2014). The majority of the silent or poorly expressed BGCs discovered so far either fail to express or show poor expression under standard fermentation conditions. Hence, there is a dire need to activate silent BGCs and enhance strain performance to promote product development and commercialization, enabling further investigation of the “biosynthetic dark matter” (Baltz 2016). In light of the current scenario, this review aims to provide a comprehensive perspective on traditional and state‐of‐the‐art strategies for optimizing the potential of *Streptomyces* strains, ultimately paving the way for enhanced production of valuable specialized metabolites.

## 2 Secondary Metabolism in *Streptomyces*—Regulatory Cascade

**Figure 1:**
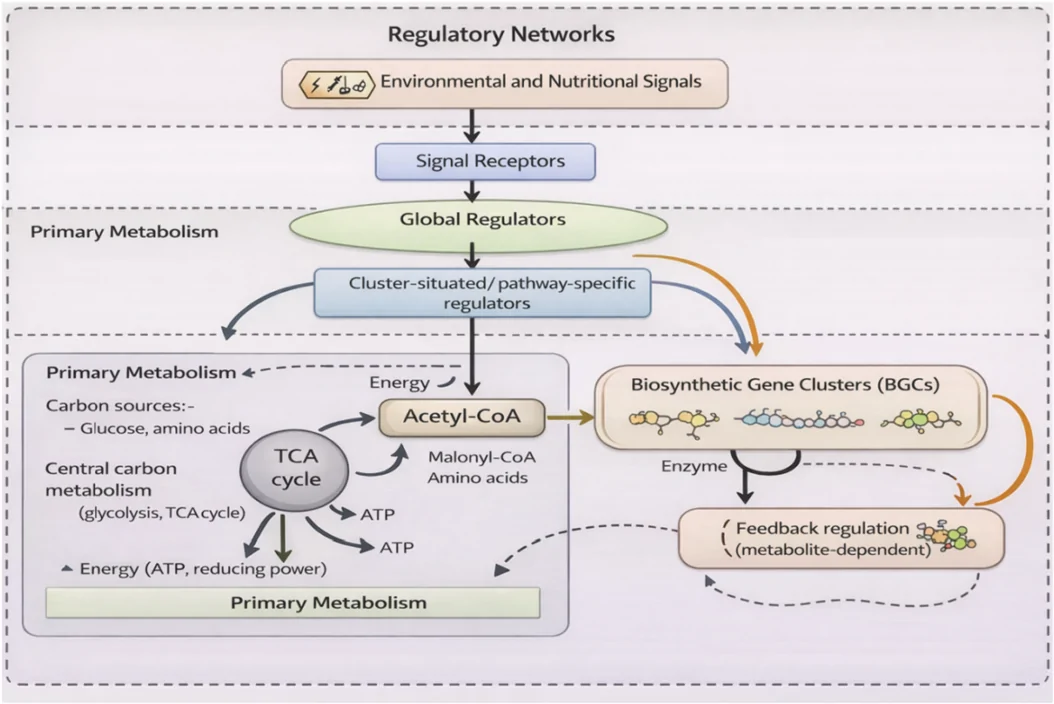
Regulatory integration of primary metabolism and secondary metabolite biosynthesis. acetyl‐CoA, acetyl coenzyme A; ATP, adenosine triphosphate; TCA, tricarboxylic acid.

SM production is often linked to the growth cycle of *Streptomyces*, particularly the switch from the vegetative state to the sporulation phase. In many instances, the synthesis of SMs typically begins during the late exponential or early stationary phase of growth. Recent studies further confirm that antibiotic biosynthesis in *Streptomyces* is tightly coordinated with morphological differentiation and is generally activated as the culture transitions from active growth to nutrient‐limited conditions (Jones 2023). Nutritional status, particularly phosphate, nitrogen, and carbon availability, is a major determinant of secondary metabolism in *Streptomyces*, often triggering antibiotic production under nutrient limitation rather than stress alone (Krysenko and Wohlleben 2024). Developmental regulators control the transition from vegetative growth to sporulation and can indirectly influence SM production by controlling the developmental processes that precede it. SM production in *Streptomyces* is controlled by hierarchical regulatory cascades consisting of environmental signal‐sensing systems, global regulators, cluster‐situated regulators (CSRs), and feedback control mechanisms that collectively coordinate BGC expression (Yan and Xia 2024) (Figure 1).

First of all, *Streptomyces* hormones/signals bind to their respective receptors and trigger a cascade of SM production pathways. One of the hormones, a factor—γ‐butyrolactone (GBL), was the first identified signal in 1967 associated with the production of Streptomycin (StrR) from *Streptomyces griseus* (Xia et al. 2020). Another study discovered avenolide, a novel type of butenolide hormone, which plays a key role in regulating avermectin, an SM, production in *Streptomyces avermitilis* (Kitani et al. 2011). Around 84.1% of actinomycetes are known to employ either GBL (64.1%) or butenolide (24%) to manage their SM production (Thao et al. 2017).

In the second tier of this cascade, global regulators influence the lower regulatory levels in multiple ways. They respond to a variety of stimuli, including nutrient shortages, fluctuations in pH, damage to the cell wall, heat shock, and the levels of *N*‐acetylglucosamine (NAG) present in the medium. These extracellular signals are detected by a two‐component system (TCS), comprising a specific regulator and an enzyme histidine kinase that monitors particular environmental cues. This TCS is the most abundant regulator of SM biosynthesis in *Streptomyces* (Thao et al. 2017). A specific TCS called PhoP/PhoR controls antibiotic production and morphological differentiation (Yepes et al. 2011; H. Rodríguez et al. 2013). In addition to regulating antibiotic biosynthesis, the PhoP/PhoR TCS coordinates phosphate homeostasis and primary metabolism, linking nutrient sensing to SM production (Sola‐Landa et al. 2003). The buildup of NAG from the autolytic breakdown of vegetative mycelium signifies the onset of secondary metabolism. DasR, a GntR family allosteric regulator, activates the production of SMs (Rigali et al. 2008) and also governs the uptake of NAG via a phosphotransferase system, in which NAG functions as a ligand (Fillenberg et al. 2016). AdpA, a member of the AraC/XylS regulatory family, participates in the transcription of a vast array of genes linked to the production of SMs. AdpA is universally distributed in *Streptomyces* to control the synthesis of metabolites (Higo et al. 2012; Guyet et al. 2014). Recent studies have demonstrated that sulfane sulfur signaling can modulate the activity of AdpA, thereby linking cellular redox metabolism with regulation of SM biosynthesis (Lu et al. 2022). Another key pleiotropic regulator is BldD, which acts as a master developmental regulator controlling morphological differentiation and secondary metabolism in *Streptomyces* by repressing numerous genes during vegetative growth and activating them during later developmental stages (McCormick and Flärdh 2012; Alam et al. 2022). BldD controls morphological differentiation and secondary metabolism through c‐di‐GMP‐dependent transcriptional regulation (McCormick and Flärdh 2012; Gallagher et al. 2024). Wb1A and AtrA are also recognized as global regulators of secondary metabolism. Wb1A functions as a global repressor, influencing the production of doxorubicin, tautomycetin, and daptomycin in their respective host organisms (Noh et al. 2010; J. H. Nah et al. 2012; H. J. Kim et al. 2014).

Pathway‐specific regulators are often associated with BGCs and control transcription of genes involved in a particular metabolic pathway. Many of these regulators are located within the BGCs and are therefore referred to as CSRs; however, the two terms are not strictly synonymous, as some regulators located within clusters can influence additional distant BGCs (Xia et al. 2020). The pathway‐specific regulator family encompasses several transcriptional regulator families, including *Streptomyces* antibiotic regulatory proteins (SARPs), the StrR family regulators, LAL (large adenosine triphosphate [ATP]‐binding regulators of the LuxR family), and PAS‐LuxR regulators, which directly control transcription of biosynthetic genes (BGs) within SM gene clusters (Liu et al. 2013; Martin and Liras 2012). Furthermore, certain transcriptional regulators such as TetR‐family proteins and some SARP‐like regulators are located outside BGCs but still exert significant control over secondary metabolism (Liu et al. 2013; Augustijn et al. 2025).

At the fourth level, feedback regulation is coordinated by metabolite production and transport. The concentration and transport of metabolites within the cell regulate secondary metabolism in *Streptomyces*. Research findings reveal that lindamycin from *Streptomyces globisporus* and actinorhodin (ACT) derived from *Streptomyces coelicolor* reduce AtrA activity, which governs both primary and secondary metabolic processes (S. Li et al. 2015). Feedback regulation occurs when the antibiotic or its intermediates influence the activity of regulatory proteins that control the BGC. For example, ACT produced by *S. coelicolor* can modulate regulatory networks that control its own biosynthesis, thereby maintaining balanced metabolite production (Liu et al. 2013).

The dynamic interplay between signals and regulators evidently influences the cell's metabolic state and plays a major role in regulating secondary metabolism. The concentration of essential nutrients also affects the type of regulation by regulators such as GlnR and PhoR–PhoP (Romero‐Rodríguez et al. 2018). According to Nett et al. (2009), most of the *Streptomyces* contain around 20 secondary metabolic pathways. Detailed studies on the SM pathway mechanism in *Streptomyces* revealed that interweaved pathways either contribute towards SM production or compete against it in favor of another metabolite (Xia et al. 2020). As an example, the AveR regulator in *S. avermitilis* facilitates the production of avermectin but simultaneously inhibits oligomycin biosynthesis (Guo et al. 2010).

Considering the intricate understanding of regulatory cascades in *Streptomyces*, various strategies and techniques can be employed to boost their biopotential. A few of these strategies are discussed below.

## 3 Key Approaches to *Streptomyces* Strain Improvement

The advent of synthetic biology has expanded the molecular tools for pathway engineering, genome editing, and the activation of cryptic BGCs (N. Lee et al. 2019; Palazzotto et al. 2019). Recent advances, including Cluster Regulatory Interspaced Short Palindromic Repeats (CRISPR)‐based systems, promoter engineering, and heterologous expression platforms, have strengthened strain improvement strategies for enhanced metabolite production (Mitousis et al. 2020; N. Lee et al. 2023; Krysenko 2025). Additionally, regulatory and nutritional factors such as nitrogen‐containing compounds are increasingly recognized as key modulators of secondary metabolism, providing further opportunities for rational metabolic engineering (Krysenko 2023). These collective advances provide the conceptual and technological framework underpinning the targeted strategies discussed in the following section.

### 3.1 Classical Strain Improvement

#### 3.1.1 Chemical and Physical Mutagenesis

Mutagenesis is the first robust method for developing efficient strains for SM synthesis. Mutagenesis is of two types: (1) chemical mutagenesis using *N*‐methyl‐*N*′‐nitro‐*N*‐nitrosoguanidine (MNNG), ethyl methanesulfonate (EMS), or nitrous acid (NA) and (2) physical mutagenesis using ultraviolet (UV) lights or X‐rays. Over the past few decades, there is not a comprehensive understanding on the factors contributing to mutagenesis in *Streptomyces*. Most efficient mutagens, that is, MNNG and EMS cause GC to AT transition mutations along with all four transversions (Baltz 1998, 2014).

Eli Lilly and Company commenced studies on mechanisms of mutagenesis in *Streptomyces* for strain improvement (Stonesifer and Baltz 1985; Baltz and Stonesifer 1985a, 1985b). Their studies showed that, similar to *Escherichia coli*, the bacteria *Streptomyces fradiae* also possess adaptive mechanisms in response to MNNG through error‐free and error‐prone DNA repair pathways. Moreover, *recA* in *S. fradiae* shows complementary functions to *E. coli recA* gene (Aparicio et al. 2000). Further studies on *Streptomyces* genomes revealed *mutT* homologs to *E. coli mutT*. *mutT* homologs are highly conserved within the actinomycetes genus. Studies on *mutT* mutator strains of *E. coli* revealed high rates of mutation via the AT–CG transversion pathway, a pattern that may extend to actinomycetes. Antibiotic resistance markers can be used to monitor mutation frequencies across various mutagens. These markers include resistance to StrR in *rpsL* genes, Rifampicin (RifR) in *rpoB* genes, and Spectinomycin (SpcR) (Baltz 2014). Recent work has demonstrated that disruption of the noncanonical mismatch repair protein NucS significantly increases spontaneous mutation rates in *Streptomyces*, highlighting the importance of DNA repair systems in generating genetic diversity that may be exploited during strain improvement programs (Dagva et al. 2024).

A study was conducted to increase the titers of avermectin B1b from *S. avermitilis* 41445 through a combination of chemical and physical mutagenesis, using UV radiation, ethidium bromide, and EMS as mutagens. The *S. avermitilis* strain exposed to UV radiation for 45 min produced the highest yield (254.14 mg/L) compared with other treatment methods (Siddique et al. 2014). In another study, UV irradiation, physical mutagenesis, and chemical mutagenesis with MNNG were used to increase rapamycin (Rap) titers produced by *Streptomyces hygroscopicus*. Mutants obtained through MNNG were found to be superior, yielding 67% higher yields than the wild‐type strain (Dutta et al. 2017).

These studies demonstrate that mutagenesis remains a convenient and feasible technique for enhancing bacterial product yield. Moreover, whole‐genome sequencing has enabled comparative studies of wild‐type strains and selected mutants to increase SM yield. This study can be coupled with omics approaches to further improve SM titers.

#### 3.1.2 Transposon Mutagenesis

Several advancements in transposon‐based mutagenesis have been made to enhance SM production. Initially, transposons *Tn5096* and *Tn5099* were used to increase SM yields, but they have certain limitations, including nonrandom target specificity and inadequacy for full‐genome analysis (Kieser et al. 2000). These constraints extend to other actinomycete transposons as well.

X. Zhang et al. (2012) developed an improved delivery system using the IS204 transposon from *Nocardia asteroides* for *S. coelicolor*. This system enabled the establishment of a library of 8000 mutants featuring diverse genes that were randomly inserted via conjugation with the transposon *pDZY101* from *E. coli*. Notably, the transposon system included a transposase gene under the ermEp promoter, an RP4 *oriT* origin of replication for *E. coli*, and antibiotic resistance genes (AmR, KanR). Among the 25 mutants with disrupted undecylprodigiosin (Red) production, one had an insertion in the *rrdA* gene, a negative regulator of *redD*, this led to increased Red production as a result of the heightened expression of the disrupted *rrdA* gene (X. Zhang et al. 2012). Beyond identifying BGs, transposon mutagenesis has also been widely used to uncover regulatory networks governing antibiotic production, enabling the discovery of previously unknown regulatory circuits controlling secondary metabolism in *Streptomyces* (Ahmed et al. 2017).

This method's high efficiency and ability to perform random insertions make it valuable for identifying regulatory genes not directly linked to SMGCs.

Further advancements involved the development of a hyperactive *Tn5* transposase system, optimized for *Streptomyces* (Petzke and Luzhetskyy 2009). Researchers engineered five specific mutations in *Tn5* transposase to create a highly efficient mini‐Tn5 system, resulting in a library of 50,000 mutants. This approach identified 551 genes affecting ACT production, many of which were previously unknown (Z. Xu et al. 2017, 2019). Moreover, a temperature‐sensitive conjugation plasmid vector featuring a thiostrepton‐inducible promoter (*tipAp*) and an R6Kgamma replicon enabled effective transposition, achieving high rates in 98% of *S. coelicolor* transconjugants. This vector showed a preference for transposing into coding regions rich in G + C content (Petzke and Luzhetskyy 2009).

A Himar1‐based synthetic transposon system was introduced, offering new delivery methods and features, including temperature‐sensitive replicons, suicide vectors, multiple antibiotic resistance options, and gene excision systems (Bilyk et al. 2013). This system demonstrated random DNA insertion in both *S. coelicolor* and *Streptomyces albus J1074*. These advancements in transposon systems provide powerful tools for genome‐wide mutagenesis, with the potential to uncover critical genes and pathways that regulate SM production in *Streptomyces*.

One promising approach involves the use of mobile group II introns, which function as retrotransposons that employ catalytic intron RNAs and intron‐encoded reverse transcriptase to facilitate precise DNA insertion via retrohoming. A recent study introduced a novel group II intron‐based gene‐editing tool specifically designed for precise chromosomal gene insertion in *Streptomyces*. By suppressing the RecA‐dependent homologous recombination pathway, the researchers improved the site‐specific insertion efficiency to 2.38%. This tool was utilized to identify and inactivate a BGC responsible for red pigment synthesis in *Streptomyces roseosporus*, resulting in enhanced cell growth and increased daptomycin production. Additionally, it was used to activate a silent jadomycin BGC in *Streptomyces venezuelae*, demonstrating its potential to unlock cryptic biosynthetic pathways. This study underscores the value of innovative genetic tools for strain improvement, SM biosynthesis, and the discovery of cryptic NPs in *Streptomyces* species (Sang et al. 2024).

#### 3.1.3 Screening for High‐Yielding Mutants

##### 3.1.3.1 Antibiotic Resistance Selection

Selecting for antibiotic resistance is a straightforward, fast, and cost‐effective method that does not require complex systems. It allows screening fewer than 100 mutants to examine potential mutations in genes, such as *rpoB* or *rpsL*. This method can detect a broad spectrum of spontaneous mutations, including base‐pair substitutions, deletions, and insertions, and avoids complications from chemically induced mutations, such as secondary negative mutations (Baltz 2011).

##### 3.1.3.2 Mutations in rpoB

Various recent studies have highlighted the potential of mutations in the rpoB gene, which encodes the β‐subunit of RNA polymerase, to improve the metabolic performance of *Streptomyces* strains. Certain mutations in the *rpoB* gene that confer resistance to Rifampicin (RifR) have been found to enhance the production of antibiotics in *Streptomyces lividans*. These include actinorhodin (Act), undecylprodigiosin (Red), and calcium‐dependent antibiotic (H. Hu et al. 2002; Lai et al. 2002; Ochi 2007). These mutations might mitigate the adverse effects of other mutations, such as those in *relA* or *rplK*, on Act production. This might happen by producing RNA polymerase β‐subunits that mimic the enzyme's ppGpp‐bound state, which is crucial for starting secondary metabolic processes (Matsushima and Baltz 1986; J. Xu et al. 2002). Spontaneous *rpoB* mutations introduced into *Streptomyces albidoflavus J1074* resulted in diverse RifR resistance profiles and substantial modulation of antibiotic production, as evidenced by bioassays and LC‐MS analysis. Such findings underscore the utility of *rpoB* mutations as a genetic tool for strain improvement, reinforcing the importance of multidisciplinary approaches—spanning genetics, biochemistry, and analytical microbiology—in unlocking the bioactive potential of *Streptomyces* (Dolya et al. 2023).

##### 3.1.3.3 Mutations in rpsL

Mutations in *rpsL* resulting in K88E or K88R substitutions can significantly increase SM production. For instance, the K88E mutation observed in *S. coelicolor* enhanced Act production from 2 to 70 mg/L, while similar mutations in *S. antibioticus* and *Streptomyces parvulus* also resulted in enhanced production (Tanaka et al. 2009). In *S. avermitilis*, the K88R mutation had negligible effect, whereas the K88E mutation resulted in lower avermectin production alongside higher oligomycin production. The production of A54145 in *S. fradiae* mutants was 371 mg/L for those with *rpsL* (K88E) and 472 mg/L for those with *rpsL* (K88R), indicating varying outcomes across the different strains (Baltz 2011).

In *S. albus*, the *rpsL* mutant KO‐600 (K88R) significantly boosted salinomycin output to 15 g/L, whereas the wild‐type strain produced only 250 mg/L. This change is attributed to heightened ribosomal activity during the stationary phase (Baltz 2011).

##### 3.1.3.4 Augmented Production of Ribosome Recycling Factor (RRF)

Overexpressing the *frr* gene, which encodes RRF, has been shown to boost Act and avermectin production. In *S. coelicolor*, the introduction of frr into a high‐copy plasmid resulted in a 7–10 times boost in Act production. In a comparable manner, *frr* overexpression from a multicopy vector in *S. avermitilis* significantly enhanced avermectin production (Hosaka et al. 2006; L. Li et al. 2010). The stable integration of *frr* could support improved fermentation processes.

##### 3.1.3.5 Combinations of Antibiotic Resistance Mutations

Combining mutations conferring resistance to StrR, RifR, and geneticin (GenR) in *S. coelicolor*, led to a 3.5‐times boost in Act production. The triple mutant strain, SGR‐1 showed improved Act production along with a prolonged production period, characterized by higher expression levels of the regulatory protein ActII‐ORF4 and decreased sensitivity to changes in the media (H. Hu and Ochi 2001). A strain known as C7, which has gained extra antibiotic resistance, generated 1.63 g/L of Act, representing a considerable rise from 9 mg/L produced by the wild‐type strain. This increase was linked to higher ppGpp levels and robust protein synthesis during fermentation (G. Wang et al. 2008). Combining RifR and StrR mutations has also been used to activate dormant antibiotic biosynthetic pathways. *Streptomyces mauvecolor* mutants with these mutations produced new antibiotics, like piperidamycin (Hosaka et al. 2009).

### 3.2 Genetic Engineering

Genetic engineering for strain improvement focuses on modifying microbial strains to boost their metabolic functions for biotechnological purposes. The goals include amplifying specific genes, increasing the production of particular proteins or products, and transferring genes between organisms. Techniques used in strain improvement involve employing DNA sources, vectors, host cells, and metabolic engineering methods. This approach finds applications in various fields, including food technology, agriculture, microbiology, and industry, where it helps enhance industrial strains. Recent studies further emphasize the expanding role of genetic engineering in improving SM biosynthesis in *Streptomyces* species. Targeted pathway modification, regulatory gene manipulation, and precursor supply optimization have been successfully employed to enhance metabolite yields and activate silent gene clusters (Kumar and Sharma 2025; Sharma et al. 2026). For instance, integrated genetic and process optimization strategies have significantly improved tacrolimus production in *Streptomyces tsukubaensis*, demonstrating the effectiveness of combining metabolic engineering with fermentation refinement (J. Zhang et al. 2025). Moreover, the development of engineered CRISPR‐Cas9 systems tailored for *Streptomyces* genome editing has enabled precise and efficient modifications to boost specialized metabolite production (D. G. Kim et al. 2025). Collectively, these advances highlight the translational potential of modern genetic tools for strain improvement and industrial‐scale bioprocess development. Various techniques used to genetically modify *Streptomyces* to enhance its potential (Table 1) are discussed below.

**Table 1:** Genetic modification of *Streptomyces* to enhance its potential.

Organism	Targeted regulatory gene	Product	Yield	References
Gene overexpression				
*Streptomyces fradiae*	*TylR*	Tylosin	75%–110% increase in production	Bate et al. (2006)
*Streptomyces avermitilis*	*pldR*	Pladienolide	Increased production	Komatsu et al. (2010)
*S. avermitilis*	*ccaR*	Cephamycin C	Increased production	Komatsu et al. (2010)
*S. avermitilis*	*strR*	Streptomycin	Increased production	Komatsu et al. (2010)
*Streptomyces albus J1074*	*tioA*	Thiocoraline	Increased production	Lombó et al. (2006)
Gene knockout and downregulation				
*S. fradiae*	*TylQ*	Tylosin	Enhanced yield	Stratigopoulos and Cundliffe (2002)
*Streptomyces platensis*	*ptmR1*	Platensimycin	323 mg/L production	Smanski et al. (2009)
*S. platensis*	*ptmR1*	Platencin	255 mg/L production	Smanski et al. (2009)
*Streptomyces coelicolor*	*scbR2*	Novel pigment and antibiotic activities	New activities, reduced actinorhodin production	Gottelt et al. (2010)
Combinatorial regulation strategies				
*Streptomyces reseiscleroticus*	*SrcmRI*	Tryptomycin	Elevated production	Sun et al. (2018)
*S. reseiscleroticus*	*SrcmRII*	Tryptomycin	Elevated production	Sun et al. (2018)
*Streptomyces carbomycin*	*AcyB2*	Carbomycin	High yields achieved	Zhong et al. (2017)
*S. carbomycin*	*CbmR*	Carbomycin	Suppressed production with overexpression of AcyB2	Zhong et al. (2017)
*Streptomyces clavuligerus*	*ccaR*	Clavulanic acid	43% increase in production	Cho et al. (2019)
*S. clavuligerus*	*claR*	Clavulanic acid	43% increase in production	Cho et al. (2019)
*Streptomyces cinnamonensis*	*MonH, MonRI, MonRII*	Monensin	Increased production through a synergistic cascade	Tang et al. (2017)
*Streptomyces mangrove Xiamen 318*	*Sxim22880, CVNABCSX, WblASX*	Polycyclic tetramate macrolactam	24.5% increase in production	Bu et al. (2020)
*S. avermitilis*	*Atr32*	Atratumycin	1.7–2.3‐fold increase in production	Z. Yang et al. (2019)

#### 3.2.1 Gene Overexpression

The overexpression of positive regulatory genes to enhance SM yield is a well‐established strategy, supported by numerous studies (Baltz and Hosted 1996; M. J. Bibb 2005; M. Bibb and Hesketh 2009; Cundliffe 2008; Olano et al. 2008). This approach has been validated by recent examples (Baltz 2010; Y. Chen et al. 2010). A key improvement is the implementation of heterologous promoters, which allows for the dissociation of pathway regulation from complicated regulatory networks, as evidenced in tylosin biosynthesis. In this instance, the TylP c‐butyrolactone receptor regulates *tylU* and *tylR* expression by repressing *tylQ* and *tylS* transcription. The elimination of *tylS* or *tylR* leads to a total loss of tylosin production, while disruption of *tylU* causes a reduction in production (Bate et al. 2006). The introduction of an extra *tylR* copy under the ermE* promoter in various strains resulted in a 75%–110% enhancement in tylosin production, with similar increases observed when additional copies of *tylS* or *tylR* were introduced (Stratigopoulos et al. 2004).

This strategy has recently been applied to other biosynthetic pathways, including the pladienolide gene cluster in *S. avermitilis* (Komatsu et al. 2010), the production of cephamycin C in *S. avermitilis* (Komatsu et al. 2010), StrR synthesis in *S. avermitilis* (Komatsu et al. 2010), fredericamycin production in *S. lividans* (Y. Chen et al. 2008), and the expression of the thiocoraline gene cluster in *S. albus* J1074 (T. Chen et al. 2008). Recent studies have highlighted the importance of pleiotropic regulatory proteins that globally influence secondary metabolism in *Streptomyces*. For example, the ATP‐dependent protease Lon (Yilmaz et al. 2025) has been shown to regulate antibiotic biosynthesis and stress responses by controlling the stability of key regulatory proteins. Similarly, the nucleoid‐associated protein Lsr2 (Ramirez et al. 2025) functions as a global transcriptional repressor that silences cryptic BGCs, and its manipulation can activate otherwise silent SM pathways. These instances confirm that leveraging strong, constitutive promoters to drive positive regulatory genes specific to pathways can significantly increase production. Notably, the case of thiocoraline demonstrates the potential of expressing SM pathways from nonstreptomycete actinomycetes in a streptomycete host, expanding the possibilities for exploring rare actinomycetes' genomes (Stratigopoulos et al. 2004).

Further advancements in strain improvement include the development of new integrative vectors with strong constitutive promoters, such as kasOp*, hrdBp, *SCO5768p*, and *SP44*. These vectors, tested in various mangrove‐derived *Streptomyces* strains, have been shown to enhance antibiotic yields by approximately 1.1–1.4 times when applied to increase the production of specific antimicrobial compounds, such as elaiophylins, azalomycin Fs, and armeniaspirols (M. Zhao et al. 2024). This integration underscores the effectiveness of combining strong promoters with gene overexpression to boost SM production in *Streptomyces*.

A recent study exemplified the potential of multidisciplinary approaches to strain improvement by significantly enhancing tylosin production in *Streptomyces xinghaiensis*. By employing a combinatorial strategy, the researchers overexpressed key genes, including the efflux gene *tlrC*, the *S*‐adenosylmethionine (SAM) synthetase gene *metKcs*, SAM BGs (*adoKcs–metFcs*), and the pathway‐specific activator *tylR*. This approach led to a remarkable increase in tylosin yield, reaching 11.35 g/L, highlighting the critical role of SAM biosynthesis and regulatory gene manipulation in optimizing SM production. Such strategies underscore the value of integrating genomic insights, metabolic engineering, and regulatory pathway activation to unlock the bioactive potential of *Streptomyces*. These findings align with the broader context of strain improvement, offering new directions for enhancing the production of pharmaceutically significant compounds in industrial strains (Dai et al. 2023).

#### 3.2.2 Gene Knockout and Downregulation

The strategies for enhancing SM production by targeting negative regulatory genes in *Streptomyces* have been widely used. In their study on *S. fradiae* C4, Stratigopoulos and Cundliffe (2002) identified an important mutation in the *tylQ* gene within the tylosin BGC that correlates with higher tylosin yields. This mutation, located in a critical DNA‐binding region, was also independently found by Y.‐X. Zhang et al. (2002) through genome shuffling. The original *tylQ* mutation likely predated the mapping and analysis of the tylosin gene cluster, highlighting the efficacy of empirical chemical mutagenesis in identifying key targets.

Recent examples of enhancing metabolite production through negative gene disruption include the work on *Streptomyces platensis*, where disruption of the *ptmR1* gene led to increased production of platensimycin and platencin, reaching 323 and 255 mg/L, respectively (Smanski et al. 2009). Another study involved the deletion of *scbR2* in *S. coelicolor*, which led to the formation of a new yellow pigment and the emergence of novel antibiotic activities alongside reduced production of the PK ACT. This suggests that deleting negative regulators can activate cryptic BGCs, potentially revealing new metabolites (Gottelt et al. 2010).

The investigation found that deletion of the *PepX* peptide gene in *S. venezuelae* had limited effects on the secondary metabolome but resulted in downregulation of secondary metabolite biosynthetic gene clusters (SMBGCs), similar to the effects of ethanol shock. Furthermore, a negative correlation was observed between the expression of certain extracytoplasmic function sigma factors (ECFs) and SMBGCs. When three specific ECF‐coding genes, which were upregulated by both *PepX* deletion and ethanol shock, were individually deleted, there was a significant boost in SMBGC expression and an increase in the production of specific SMs. Notably, the deletion of one ECF‐coding gene in a *Streptomyces* sp. derived from marine sponges altered its SM profile, emphasizing the critical regulatory role of ECFs in secondary metabolism (Sekurova et al. 2024). Using bioinformatic tools, these approaches could be applied to other orphan or cryptic gene clusters identified through genome analysis.

##### 3.2.2.1 Combinatorial Regulation Strategies

Achieving higher yields of target products is effective when positive regulators are upregulated while negative regulators are downregulated simultaneously. For example, in *Streptomyces reseiscleroticus*, an increase in tryptomycin production was achieved by overexpressing the activator *SrcmRI* and disrupting the inhibitor *SrcmRII*, which together led to elevated tryptomycin levels (Sun et al. 2018). Similarly, in *Streptomyces cinnamonensis*, coordinating the positive regulators *MonH*, *MonRI*, and *MonRII* enhanced monensin production through a synergistic cascade, where *MonH* up‐regulates *MonRII*, which in turn enhances *MonRI* transcription (Tang et al. 2017). In another scenario, the joint overexpression of *ccaR* and *claR*, both positive regulatory genes, led to a 43% improvement in clavulanic acid production in *Streptomyces clavuligerus* OR (Cho et al. 2019). Additionally, overexpressing the regulator *AcyB2* and knocking out the inhibitor *CbmR* significantly boosted carbomycin yields (Zhong et al. 2017), while overexpressing *Sxim22880*, *CVNABCSX*, and *WblASX* resulted in a 24.5% increase in polycyclic tetramate macrolactam production in *Streptomyces mangrove* Xiamen 318 (Bu et al. 2020). Furthermore, the titers of atratumycin in *Streptomyces* were improved around 1.7–2.3‐fold by rationally engineering regulatory genes and transporters, including the negative regulator *Atr32* (Z. Yang et al. 2019).

Doxorubicin, an anthracycline antitumor antibiotic produced by *Streptomyces peucetius*, holds significant therapeutic value. However, the wild‐type strain's low fermentation yield makes direct industrial‐scale production economically unfeasible. In this study, an improved strain, *S. peucetius* SIPI‐7‐14, was developed from SIPI‐14 through repeated rounds of doxorubicin resistance screening. To enhance production, the ketoreductase gene (*dnrU*) was knocked out to suppress the formation of (13S)−13‐dihydrodaunorubicin, a competing byproduct, while overexpressing the resistance gene (*drrC*) improved the strain's tolerance to doxorubicin. The engineered strain, *S. peucetius* ΔU1/*drrC*, achieved a doxorubicin yield of 1128 mg/L, representing a 102.1% increase compared with SIPI‐14. Further optimization of the fermentation medium using the response surface method resulted in a yield of 1406 mg/L in shake‐flask cultures after 7 days. Scaling up to a 10‐L fermenter, the yield increased to 1461 mg/L, the highest reported to date, highlighting the strain's potential for cost‐effective industrial‐scale production of doxorubicin (S. Yang et al. 2024).

#### 3.2.3 Genomic Duplication of SM Biosynthetic Pathways

Increasing the number of genes responsible for the rate‐limiting processes in antibiotic production has effectively improved the overall yield of antibiotics (Baltz and Hosted 1996; Guo et al. 2010; Baltz 2001; Olano et al. 2008). Amplifying entire SM BGCs offers dual benefits: it directly increases product yield and improves the production of essential precursors and cofactors through mutagenesis or metabolic engineering. Industrial strain improvement programs have seen some success with whole‐pathway duplications or higher‐order amplifications. As an example, the oxytetracycline BGC in *Streptomyces rimosus* is located within a genetically unstable region approximately 600 kb from the chromosome terminus. Variants with multiple copies of the gene cluster or its associated plasmid showed increased oxytetracycline production, though their instability limited their commercial use (Petković et al. 2006). In a similar study of the lincomycin gene cluster in a strain of *Streptomyces lincolnensis* used for producing lincomycin, a duplicated DNA region between 450 and 500 kb was found (Peschke et al. 1995). Likewise, *Streptomyces kanamyceticus* showed a threefold average DNA amplification of a 145‐kb segment that harbors the kanamycin gene cluster (Yanai et al. 2006). The instability of these amplifications, which were not preserved during sporulation, necessitated maintaining strains as vegetative mycelia.

New advancements make it possible to clone large DNA segments into bacterial artificial chromosome (BAC) vectors, thereby facilitating the controlled replication of important SM gene clusters while preserving their functionality (Alexander et al. 2010; Baltz 2009; Baltz et al. 2010; Z. Feng et al. 2009). Using various attachment and integration systems of streptomycete bacteriophage like /C31, /BT1, TG1, and R4, these gene clusters can be stably inserted into bacteriophage attachment sites (attB) (Bierman et al. 1992; Kuhstoss and Rao 1991; Smith et al. 2010; Gregory et al. 2003; Morita et al. 2009; Shirai et al. 1991). These systems take advantage of attB sites found in robust core regions of linear Streptomycete chromosomes, compared with the less secure ends that harbor a variety of cryptic SM gene clusters. For instance, inserting a cosmid clone containing moenomycin BGs into the /C31 bacteriophage attachment site (attB) of *Streptomyces ghanaensis* resulted in a twofold increase in moenomycin production (Makitrynskyy et al. 2010). Additionally, removing the lipopeptide A54145 BGC from a moderately productive *S. fradiae* strain and integrating it into either the /C31 or /BT1 sites boosted production levels to 513 and 583 mg/L, respectively. This method relocated the gene cluster from an unstable region to a more stable core region of the chromosome. Such strategies for duplicating or triplicating cryptic gene clusters in stable chromosomal regions can facilitate the exploration of poorly expressed or cryptic pathways and further boost product yields (Alexander et al. 2010).

#### 3.2.4 Targeting Competing Pathways for Enhanced SM Production

In principle, actinomycetes that generate multiple SMs might encounter competition for essential precursors, cofactors, energy sources, reducing agents, and other vital resources, which could limit the production of the most sought‐after compounds. A notable example is *S. avermitilis*, known for synthesizing various large PKs, such as avermectin and oligomycin, along with other SMs (Ikeda et al. 2003). Komatsu et al. (2010) engineered a genome‐minimized variant of *S. avermitilis* by removing several SMGCs. This alteration significantly improved the heterologous expression of the StrR BGCs from *S. griseus*, raising production levels from roughly 30 mg/L in the wild‐type strain to about 180 mg/L in the altered strain. Additionally, other hosts for heterologous expression have been created by knocking out or inactivating one or more essential SM pathways (Baltz 2010).

### 3.3 Awakening Dormant BGCs

*Streptomyces*, famous for its significant biosynthetic prowess, produces more than 70% of all natural scaffolds used to obtain antimicrobials. The genomes of *Streptomyces* species typically contain 25–50 BGCs (L. Li et al. 2021); however, nearly 90% of these clusters remain silent or cryptic in conventional laboratory settings. This poses a substantial challenge to uncovering novel SMs or increasing the output of existing compounds. Consequently, recent efforts have focused on strategies to activate these dormant BGCs to boost SM production (H. Ren et al. 2020). The techniques involve (1) taking advantage of extracellular signals, (2) restructuring SM biosynthetic pathways in their native hosts, and (3) employing heterologous expression to awaken silent BGCs (Z. Xu et al. 2022).

#### 3.3.1 Activation of Silent BGCs by Extracellular Signals

Environmental conditions and nutrient levels can greatly influence NP production in *Streptomyces*. A promising method for enhancing NP yields or discovering new compounds involves the use of extracellular signals, which encompass environmental stressors and hormone‐like molecules. Stressors like heat shock, alterations in pH, and high salinity levels have been shown to stimulate silent BGCs and promote SM production. For instance, heat shock, which triggers a broad cellular response to elevated temperatures, can enhance NP yields; a study demonstrated that treating *Streptomyces* spore suspensions at a temperature of 50°C for a time duration of around 10 min increased actinomycin C yields by 27% (H. Lu et al. 1999). Correspondingly, the application of pH shock facilitates improved proton transport and cell respiration, thereby elevating ATP production. Research indicates that a 27.43% increase in validamycin A can be seen by exposure to alkaline pH shock in *S. hygroscopicus* 5008 (J. Jiang et al. 2018).

The small, diffusible compounds, referred to as hormone‐like signaling molecules, are crucial in activating global regulators that drive the transcription of genes associated with secondary metabolism and morphological differentiation. Over 14 GBLs are known to self‐regulate in approximately 60% of *Streptomyces* species. For instance, the introduction of the GBL analog 1,4‐butyrolactone to the culture of *Streptomyces* producing validamycin A led to a 30% increase in the titers of the antibiotic in both shake flasks and bioreactors (G. Y. Tan et al. 2013). Also, avenolide compounds are vital for the activation of avermectin production; four different such compounds identified in *S. albus* were applied to significantly enhance the yield of novel avermectin derivatives in *S. avermitilis* (T. B. Nguyen et al. 2018).

#### 3.3.2 Reconfiguration of BGC Pathways in Native Hosts

An alternative method for triggering inactive BGCs is to reconfigure their biosynthetic pathways. However, this method faces challenges due to the complex regulatory mechanisms governing BGCs. To address these challenges, researchers have employed the technique of engineering promoters and ribosomes. Promoter elements play an important part in promoting efficient transcription, which is crucial for reactivating silent genes. They can be categorized as either constitutive or inducible types. For example, the genomic analysis of *S. coelicolor M145* revealed the presence of a strong constitutive promoter, *Psco5768*, which has been shown to enhance the production of jadomycin by doubling its titer when used to drive the jadomycin BGC (S. Li et al. 2015). In contrast, the constitutive promoter in *S. coelicolor* M145, which produces ACT, was substituted with a cumate‐inducible promoter, which led to a threefold increase in ACT titers under optimal induction conditions, that is, time duration of 35 h and 2.5 µM cumate concentrations (S. Li et al. 2018). This highlights that fine‐tuning the balance between product biosynthesis and other physiological functions via inducible promoters can significantly increase yields in *Streptomyces*.

X. Zhao et al. (2023) employed the CRISETR method, a powerful gene‐editing tool that combines the CRISPR/Cas9 system with RecET‐mediated recombination for precise and multiplexed modifications in BGCs. This technique enables the targeted modification of promoter sites within native BGCs, providing a novel approach to activating silent pathways and optimizing metabolite production. Applied to *S. coelicolor* A3(2), the CRISETR method led to a remarkable 20.4‐fold increase in daptomycin production through promoter modifications. This study highlights the potential of regulatory element engineering to reconfigure BGC pathways and enhance the metabolic output of native strains, making it an invaluable tool for both strain improvement and the exploration of cryptic NPs. The CRISETR method exemplifies how precise genetic interventions can drive significant advances in SM production, offering new avenues for metabolic engineering and strain optimization.

Ribosomal engineering, encompassing techniques such as classical mutagenesis and metabolic engineering, is another powerful approach to activating dormant BGCs. Conventional mutagenesis focuses on identifying mutants that are resistant to antibiotics and exhibit increased production of the desired product (Kozo 2016). Mutations in ribosomal protein genes, such as rpsL, are frequently identified during screening of spontaneous antibiotic‐resistant mutants and can significantly enhance SM production by altering translational fidelity and global regulatory networks (Lopatniuk et al. 2019). For instance, an innovative approach to stimulate ε‐poly‐l‐lysine production combined atmospheric and plasma mutagenesis, yielding a total of six antibiotic resistance mutations. The resulting R6 mutant achieved a production boost of 4.41 g/L of ε‐poly‐l‐lysine, marking a 2.75‐fold improvement compared with the parental strain (L. Wang et al. 2018). A study on the genome of *S. lividans* TK21 led to the successful identification of a new compound, streptorubin B by overexpressing the genes of exogenous *rpsL* (ribosomal protein S12) and *rpoB* (RNA polymerase β‐subunit) genes. They employed the strategy of “Transcription and translation in one” approach to awaken the dormant BGCs, which were related to a derivative of undecylprodigiosin from the *S. lividans* TK21 genome (Z. Xu et al. 2022). In addition to spontaneous mutations, engineered rpsL mutations have also been successfully introduced through genetic engineering strategies to activate cryptic biosynthetic pathways and improve metabolite yields (Lopatniuk et al. 2019). In addition to transcriptional activation strategies, manipulation of DNA repair systems and recombination pathways has also been proposed as an effective approach for activating silent BGCs by promoting genome rearrangements and adaptive evolution (Sekurova et al. 2021).

#### 3.3.3 Employing Heterologous Expression to Awaken Silent BGCs

The strategies of cloning and heterologous expression of silent or low‐expressed SMGCs (Table 2) have been effective in enhancing yields and facilitating the isolation and analysis of new compounds (Baltz 2010; Gomez‐Escribano and Bibb 2011). This approach proves especially valuable for activating cryptic pathways in rare actinomycetes that are challenging to grow or unsuitable for large‐scale fermentation. Laboratory strains such as *S. lividans* and *S. coelicolor* have been used for this purpose, though yields tend to be modest. For instance, yields for *S. lividans* range from 0 to 400 mg/L, with a median value of 20 mg/L, while *S. coelicolor* yields fall between 23 and 47 mg/L, averaging approximately 30 mg/L (Baltz 2010).

**Table 2:** Heterologous expression of SMGCs in *Streptomyces*.

Sr. No.	Expression host	Secondary metabolite	Type of effect	Gene/pathway modified	References
1	*Streptomyces lividans*	Various	General transcriptional modification	Not specified	Baltz (2010)
2	*Streptomyces coelicolor*	Various	General transcriptional modification	Not specified	Baltz (2010)
3	*S. coelicolor* M1154	Chloramphenicol, congocidine, caprazamycin derivatives	Gene activation	rpoB mutation	Gomez‐Escribano and Bibb (2011)
4	*S. coelicolor* M1154	Caprazamycin derivatives	Gene activation	rpoB mutation	Flinspach et al. (2010)
5	*Streptomyces albus* J1074	Iso‐migrastatin	Activation of specific biosynthetic pathway enzymes for targeted metabolite production	Iso‐migrastatin biosynthetic pathway	Baltz (2010)
6	*S. albus* J1074	Fredericamycin	Activation of specific biosynthetic pathway enzymes for targeted metabolite production	Fredericamycin biosynthetic pathway	Baltz (2010)
7	*Streptomyces ambofaciens* BES2074	A54145 factors	High native transcriptional activity of the biosynthetic cluster without genetic manipulation	a54145 biosynthetic gene cluster (BGC)	Alexander et al. (2010)
8	*Streptomyces fradiae* C373.1	Hybrid polyketides (e.g., Tylosin)	Introduction of hybrid genes and rearrangement of BGCs to produce new hybrid metabolites	tylosin BGC	Cundliffe (2008) and Stratigopoulos et al. (2004)
9	*Streptomyces cinnamonensis* C730.7	Tetracenomycin	Optimization of transcription through media engineering and pathway regulation	tcm BGC	C. Li et al. (2009)
10	*S. cinnamonensis* C730.7 (monensin‐lacking strain)	Tetracenomycin	Pathway‐specific inactivation of monensin production to enhance tetracenomycin yield	Monensin biosynthetic pathway deletion	C. Li et al. (2009)
11	*Streptomyces avermitilis* SUKA17	Various	Deletion of major biosynthetic pathways to allow enhanced expression of alternative metabolites	Multiple major pathways deleted	Komatsu et al. (2010)
12	*S. fradiae* DA1187	Various	Knockout mutations to suppress negative regulators and overexpression of key biosynthetic genes (BGs) to boost production	Fradimycin BGC	Alexander et al. (2010)
13	*Streptomyces roseosporus* UA431	Various	Gene cluster deletion to suppress primary metabolite synthesis and redirect resources	Daptomycin BGC deletion	K. T. Nguyen et al. (2006)
14	*Saccharopolyspora erythraea*	Various	Targeted inactivation of erythromycin BGs to enable expression of alternative products	Erythromycin BGC	E. Rodriguez et al. (2003)

Abbreviation: SMGCs, secondary metabolite biosynthetic gene clusters.

Recent advances in synthetic biology have led to the development of specialized *Streptomyces* expression chassis strains with minimized endogenous biosynthetic pathways, enabling efficient heterologous expression and improved discovery of SMs (Lasch et al. 2026). The development of the *S. coelicolor* M1154 strain, which lacks several BGCs and contains mutations that enhance SM production, has led to significant increases in yields of chloramphenicol, congocidine, and caprazamycin derivatives (Gomez‐Escribano and Bibb 2011; Flinspach et al. 2010). This strain is expected to be effective for expressing cryptic pathways because of its streamlined secondary metabolome and increased production during the stationary phase. *S. albus* J1074 is another noteworthy microbial strain, yielding two compounds iso‐migrastatin and fredericamycin at 46 and 132 mg/L, respectively (Baltz 2010).

Expression hosts derived from industrial production strains or through mutagenesis have also shown promise. For example, cloning the biosynthetic pathway of A54145 lipopeptide antibiotic into a BAC vector and transferring it to *Streptomyces ambofaciens* BES2074 led to a fourfold increase in A54145 factor production compared with wild‐type *S. fradiae* A54145 (99). Similarly, strains of *S. fradiae* and *S. cinnamonensis* have been optimized for the production of tylosin and tetracenomycin, respectively, with significant improvements in yield through specific genetic modifications and fermentation conditions (Cundliffe 2008; Stratigopoulos et al. 2004; C. Li et al. 2009).

Other effective expression hosts include *S. avermitilis* SUKA17, *S. fradiae* DA1187, *S. roseosporus* UA431, and *Saccharopolyspora erythraea* (Komatsu et al. 2010; Alexander et al. 2010; K. T. Nguyen et al. 2006; E. Rodriguez et al. 2003). The above‐mentioned hosts have been altered to enhance SM production by either deleting native gene clusters or introducing new ones, demonstrating the versatility and potential of genetic engineering in optimizing biosynthetic pathways. Although activation of dormant BGCs primarily facilitates NP discovery, similar regulatory and genomic engineering strategies can also be applied to improve the production efficiency of already known SMs.

### 3.4 Metabolic Engineering

Genetic engineering enables researchers to manipulate specific genes within a BGC, allowing for the overexpression of key enzymes or the suppression of competing pathways. However, these targeted interventions often require complementary strategies to address system‐wide bottlenecks. Metabolic engineering extends this framework by considering the broader metabolic network, optimizing flux distributions, and minimizing energy losses. For instance, while genetic engineering can enhance the expression of a single gene involved in SM synthesis, metabolic engineering ensures the entire pathway operates efficiently to maximize yield. Together, these approaches represent complementary tools for improving *Streptomyces* strains.

**Figure 2:**
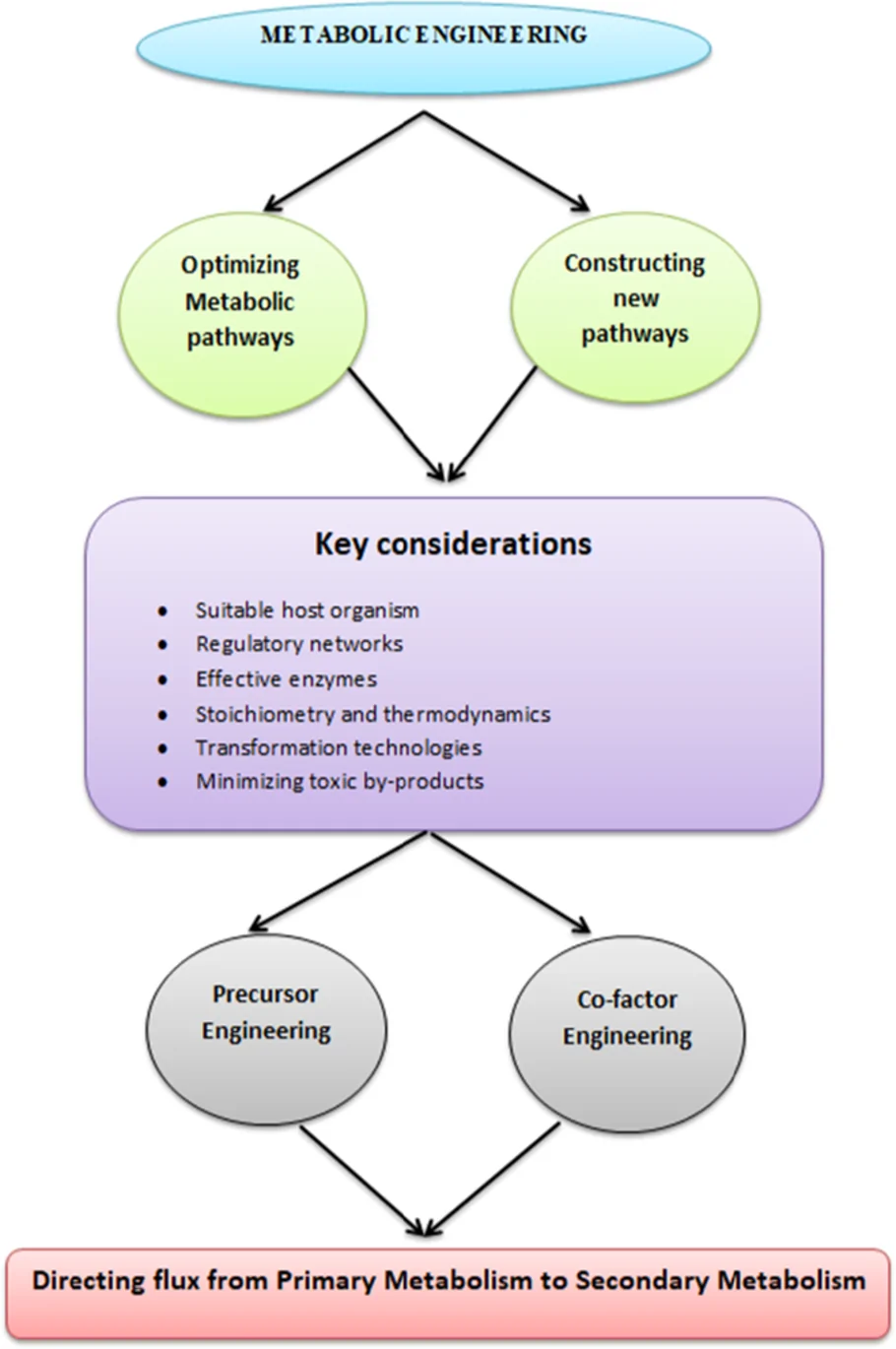
Metabolic engineering for secondary metabolite yield enhancement.

Metabolic engineering (Figure 2) aims to enhance the production of target molecules by optimizing metabolic pathways and regulatory networks, or constructing new pathways. Key considerations include choosing a suitable host organism, understanding metabolic networks, selecting effective enzymes, analyzing stoichiometry and thermodynamics, ensuring efficient transformation technologies, and minimizing toxic byproducts (Zhu and Jackson 2015).

Recent advancements in metabolic engineering have focused on redirecting carbon flux from primary to secondary metabolism. A notable study by W. Wang et al. (2020) demonstrated that triacylglycerols can be used as a carbon source for PK production in *S. coelicolor* A3(2). PKs, synthesized by polyketide synthases (PKSs), rely on acetyl‐CoA and malonyl‐CoA, which are crucial for both primary and secondary metabolic processes (Y. Chen et al. 2020; Staunton and Weissman 2001). Advances in systems‐level metabolic engineering further demonstrate the power of rational strain optimization in *Streptomyces* species. Genome‐scale metabolic modeling (GSMM) has enabled targeted interventions to enhance antibiotic biosynthesis, as illustrated by model‐guided engineering of *Streptomyces leeuwenhoekii* to significantly increase chaxamycin production (Rubio et al. 2025). GSMM integrates genomic, biochemical, and physiological information into a computational framework to predict intracellular metabolic fluxes and identify engineering targets to enhance metabolite production. Similarly, pathway redirection and metabolic flux optimization strategies have been successfully applied to improve the production of the reverse antibiotic nybomycin in *Streptomyces explomaris*, underscoring the value of integrated systems biology approaches (W. Shu et al. 2025). These studies reinforce the effectiveness of combining computational modeling with precise genetic modifications to achieve industrially relevant yield improvements. Moreover, coordinated regulation of carbon, nitrogen, phosphate, and sulfur metabolism plays a critical role in the supply of precursors for SM biosynthesis, highlighting nutrient‐responsive metabolic networks as strategic targets for strain improvement (Krysenko and Wohlleben 2024). Together, such data‐driven and metabolism‐centered strategies provide a robust framework for future rational strain development and scalable bioprocess optimization.

Precursor Engineering has been pivotal in enhancing SM yields. For instance, manipulating carbohydrate metabolism pathways, such as the Embden–Meyerhof and pentose phosphate pathways, has led to significant increases in metabolite production. Disruption of glyceraldehyde‐3‐phosphate dehydrogenase genes in *S. clavuligerus* resulted in a 3.1‐fold increase in clavulanic acid production (R. Li and Townsend 2006). A recent example of the successful implementation of metabolic engineering strategies in a native *Streptomyces* host demonstrated enhanced FK506 production in *S. tsukubaensis* by optimizing precursor supply through targeted pathway engineering, highlighting the practical impact of integrated metabolic redesign in industrially relevant strains (Schulz et al. 2022). Similarly, targeting glucose‐6‐phosphate dehydrogenase isoenzymes in *S. lividans* enhanced the production of undecylprodigiosin and ACT (Butler et al. 2002).

Fatty Acid Precursors have also been engineered to boost PK production. For example, inactivating the methylmalonyl‐CoA mutase gene in *S. erythraea* led to increased erythromycin production under oil‐based conditions (C. D. Reeves et al. 2004; A. R. Reeves et al. 2006, 2007). In *S. avermitilis*, inactivating the branched‐chain α‐keto acid dehydrogenase gene increased oligomycin production (Cropp et al. 2001; X. Wei et al. 2006). Overexpression of acetyl‐CoA carboxylase in *S. coelicolor* resulted in a sixfold increase in ACT production (Ryu et al. 2006).

Cofactors such as SAM are crucial for secondary metabolism. Overexpression of the metK gene, encoding SAM synthetase, in *S. lividans* and *S. coelicolor* led to increased ACT production (D. J. Kim et al. 2003; Okamoto et al. 2003). Similarly, overexpression of metK1‐sp in *S. venezuelae* enhanced pikromycin production (Maharjan et al. 2008). These strategies highlight the effectiveness of metabolic engineering in enhancing SM yields by optimizing precursor availability and cofactor levels.

### 3.5 Role of Optimizing Culture Conditions

Fermentation optimization focuses on tailoring media composition, pH, temperature, and process parameters to maximize SM production. This approach complements genetic and metabolic engineering strategies, as even highly optimized strains may fail to reach their full potential under suboptimal fermentation conditions. For example, nutrient‐rich media can enhance precursor availability for biosynthetic pathways, while controlled pH and temperature conditions stabilize enzymatic activities critical for metabolite synthesis. Thus, fermentation optimization serves as a critical step in unlocking the potential of the strain. Modifying culture conditions—such as medium composition, temperature, pH, and oxygen levels—greatly influences the yield of specialized metabolites in *Streptomyces*. For example, the OSMAC (one strain–multicompound) approach, introduced by Zeeck and colleagues in the early 2000s, entails culturing target bacteria under diverse conditions and analyzing all the metabolites produced (Bode et al. 2002). This approach led to the discovery of new compounds, such as chaxalactins A–C and terrosamycins A and B (Rateb et al. 2011; Sproule et al. 2019).

Specific culture conditions have been shown to enhance SM production. High‐temperature culture and heat‐shock metabolites (HSMs)—metabolites activated by elevated temperatures—have been studied as a means to enhance metabolite production. For instance, high‐temperature cultivation can activate dormant genes and increase metabolite yields. Heat shocks at 42°C significantly boosted jadomycin B production in *S. venezuelae* ISP5230 (Doull et al. 1993). Similarly, optimized temperature conditions improved granaticin production in *Streptomyces thermoviolaceus* NCIB 10076, with optimal yields achieved at 37°C (James and Edwards 1989). High‐temperature culture conditions have been explored as a strategy to activate dormant genes in actinomycetes, potentially leading to the production of novel SMs. Saito et al. (2020) conducted an extensive study involving 3160 actinomycete strains isolated from Japanese soil, identifying 57 strains capable of growing at 45°C. The analysis of their metabolome showed that almost half of these strains produced SMs specifically at elevated temperatures. Further examination of 16 strains across different media revealed varied effects on metabolite production, suggesting that culturing at high temperatures can trigger the formation of dormant SMs, termed HSMs. Notable HSMs include Gaudimycins A and B, discovered in *Streptomyces* sp. CA40 through heterologous expression of BGCs (Palmu et al. 2007), and other metabolites like resistomycin and resistoflavin from *Streptomyces* sp. HN66 (Carlson et al. 2015).

The mechanisms underlying HSM production are being investigated, with a focus on the roles of heat‐shock proteins and reactive oxygen species. Heat stress is known to activate genes encoding heat‐shock proteins, which may influence metabolite production (Grandvalet et al. 1997; Grandvalet et al. 1998; Servant et al. 1999). Research by X. Lu et al. (2021) has shown that *HspR* regulates avermectin production in *S. avermitilis*. Further studies are needed to understand how heat‐shock sensors and reactive oxygen species affect SM production under high‐temperature conditions (Hahn et al. 2000, 2002; Dela Cruz et al. 2010; Z. H. Wei et al. 2011; Kudo et al. 2022).

Adjustments in pH also affect metabolite yields. Study on *S. hygroscopicus* 5008 resulted in enhanced production of SM known as validamycin A by giving NaOH shocks (J. Jiang et al. 2018), while acidic pH shocks enhanced ε‐poly‐ʟ‐lysine production in *Streptomyces* sp. M‐Z18 (X. D. Ren et al. 2015). Conversely, the addition of disodium hydrogen phosphate improved antarlide production by mitigating excessive acidity (Saito et al. 2016).

Oxygen supply and light exposure further influence metabolite production. Increased oxygen supply was shown to enhance pristinamycin production in *Streptomyces pristinaespiralis* (X. D. Ren et al. 2015), and light‐induced carotenoid pigment production in *S. coelicolor* A3(2) was regulated by transcription factors *LitR* and *LitS* (Takano et al. 2005).

A study highlights the potential of culture optimization and strain improvement in enhancing SM production. Researchers isolated *Streptomyces* sp. KRA16‐334, which produced a herbicidal compound (334‐W4) with significant activity against weeds. The yield of 334‐W4 was enhanced through UV mutagenesis and culture optimization, including adjustments in pH, temperature, agitation, and the addition of trifluoroacetic acid. A mutant strain (0723‐8) achieved a 2.8‐fold higher yield (264.7 mg/L) than the wild type. These findings underscore the importance of targeted optimization strategies for improving the production of bioactive metabolites in *Streptomyces* (Y. S. Kim et al. 2024).

In another study, the production of Chrysomycin A (CA), a potent antibiotic active against Gram‐positive bacteria and cancers, was enhanced through fermentation optimization in *Streptomyces* sp. 891‐B6, a UV‐induced mutant with improved CA yield. Optimal conditions included 12 days of fermentation, seed age of 5 days, 5% inoculum volume, 200 mL flask loading volume, and an initial pH of 6.5. Using response surface methodology, the ideal medium composition was identified as glucose (39.283 g/L), corn starch (20.662 g/L), soybean meal (15.480 g/L), and CaCO_3_ (2.000 g/L). These optimizations resulted in a 60% increase in CA yield (1601.9 mg/L), demonstrating the effectiveness of integrating mutagenesis and culture optimization for enhancing SM production in *Streptomyces* (Z. Hu et al. 2024).

A research found the significance of optimizing culture conditions for maximizing bioactive metabolite production in *Streptomyces*. Four promising strains (*Streptomyces spectabilis*, *Streptomyces purpurascens*, *Streptomyces coeruleorubidus*, and *Streptomyces lavendofoliae*), isolated from soil samples, and exhibited varying optimal conditions for metabolite production. For *S. spectabilis*, maximum production occurred with cellobiose and peptone on the fifth day at pH 5 and 30°C. *S. purpurascens* showed optimal production with starch and casein at pH 7, 30°C, and an 8‐day incubation period. *S. coeruleorubidus* achieved the highest yield with mannitol and JBM at pH 6 and 35°C on the 6th day, while *S. lavendofoliae* required starch and peptone at pH 7, 30°C, and a 10‐day incubation. These findings underscore the strain‐specific nature of culture optimization and its critical role in enhancing SM production in *Streptomyces* (Bundale et al. 2015).

Overall, these findings (Table 3) illustrate how manipulating culture conditions can lead to substantial improvements in the yield of SMs, highlighting the importance of optimizing these factors in microbial fermentation processes.

**Table 3:** Manipulation of culture conditions to improve the yield.

Organism used	Findings	Yield enhancement		References
Effect of regulators				
*Streptomyces coelicolor* M145	Inactivation of the Rok7B7 regulator led to twice as much increase in the production of Actinorohdin. The same regulatory change resulted in an increase in undecylprodigiosin production	Actinorohdin undecylprodigiosin	2‐fold 1.5‐fold	Swiatek et al. (2013)
*S. coelicolor* A3(2)	Manipulation of the AfsQ1/Q2 regulatory system led to a significant increase in the production of coelimycin P2	1.5‐fold		S. Chen et al. (2016)
*S. coelicolor* A3(2)	Manipulation of the AfsQ1‐Q2‐SigQ signal transduction system resulted in an increase in actinorhodin (ACT) production. It was also observed that undecylprodigiosin production increased due to the effects of the AfsQ1‐Q2‐SigQ system	Actinorohdin undecylprodigiosin	1.8‐fold 1.6‐fold	D. Shu et al. (2009)
The presence or absence of metals				
*Streptomyces aureofaciens* ATCC 10762	Iron limitation resulted in higher tetracycline yields compared with conditions with sufficient iron.	2.5‐fold		Bechet and Blondeau (1998)
*Streptomyces thioluteus*	Copper depletion resulted in higher production of SF2768 compared with conditions with adequate copper levels.	3‐fold		L. Wang et al. (2017)
*Streptomyces* sp. YB‐1	The presence of certain rare‐earth elements, particularly when ytterbium was decreased, significantly enhanced the production of naphthoquinone pigments	2.3‐fold		Kamijo et al. (1999)
*S. coelicolor* A3(2)	The presence of rare‐earth elements significantly enhanced the production of ACT and undecylprodigiosin	Actinorohdin undecylprodigiosin	2.5‐fold 2‐fold	Tanaka et al. (2010)
pH changes				
*Streptomyces hygroscopicus* 5008	Reported that the validamycin A yield increased in response to the alkaline pH shock treatment compared with the control conditions without pH shock.	2.7‐fold		J. Jiang et al. (2018)
*Streptomyces* sp. M‐Z18	Acidic pH shock resulted in an increase in ε‐poly‐l‐lysine production compared with the control conditions without pH shock	2.5‐fold		X. D. Ren et al. (2015)
Oxygen supply and light quality				
*Streptomyces pristinaespiralis*	Optimizing the oxygen supply led to an increase in pristinamycin production compared with conditions with suboptimal oxygen levels	1.8‐fold		X. D. Ren et al. (2015)
*S. coelicolor* A3(2)	Researchers identified a sigma factor that plays a critical role in light‐induced carotenogenesis. Carotenoid production increased when the bacteria were grown under light conditions compared with dark conditions	3.2‐fold		Takano et al. (2005)
High‐temperature culture				
*Streptomyces venezuelae* ISP5230	Heat shock at 42°C increased the production of jadomycin B by approximately compared with nonheat‐shocked cultures	1.7‐fold		Doull et al. (1993)
*Streptomyces thermoviolaceus* NCIB 10076	Adjusting the culture temperature to 45°C resulted in an optimal yield of granaticin, while the highest synthesis rate was observed at 37°C.	2.2‐fold		James and Edwards (1989)
*Streptomyces* sp. CA40	Identified Gaudimycins A and B as HSMs with enhanced production under high‐temperature conditions.	2.3‐fold		Palmu et al. (2007)
*Streptomyces* sp. AY2	Murecholamide was identified as an HSM with an increase in production at high temperatures.	1.8‐fold		Saito et al. (2020)
*Streptomyces* sp. *HR41*	Discovered noaoxazole with a notable increase in production under high‐temperature conditions.	3.1‐fold		Saito et al. 2022)
*Streptomyces* sp. *JA74*	Dihydromaniwamycin E production saw a 2.5‐fold increase with high‐temperature cultivation	2.5‐fold		Saito et al. (2022)
*Streptomyces* sp. *B033* and *Burkholderia vietnamiensis ATCC BAA‐248*	Resistomycin was produced with increased yield through cocultivation under high‐temperature conditions.	Specific yield improvements were not detailed, but production was enhanced		Carlson et al. (2015)

### 3.6 Adaptive Laboratory Evolution (ALE)

In laboratory settings, researchers often study pathogen resistance development by serially exposing them to increasing concentrations of antibiotics over time (Banerjee et al. 2008; Locke et al. 2009; Berger‐Bächi et al. 1989). This evolution‐based method, known as ALE, results in mutants with enhanced resistance compared with the original strains or any spontaneous mutants present at the start. ALE not only fosters adaptation to new environments but also generates various phenotypes beyond antibiotic resistance (Charusanti et al. 2012).

The goal of developing industrial strains is to enhance productivity (the volume of product generated in a given timeframe) and yield (the ratio of product output to substrate input) (Cho et al. 2019). ALE of industrial strains can shorten cycle times and enhance product titer, strain tolerance, and substrate utilization efficiency. The applications of ALE for strain improvement typically concentrate on (1) optimizing growth with specific nutrients and promoting prototrophy, (2) adapting to challenging conditions (such as suboptimal pH and temperature) or tolerating harmful environments like process‐generated inhibitors, and (3) producing new compounds or boosting product titer (S. Lee and Kim 2020).

Empirical ALE studies aimed at enhancing productivity and substrate flexibility have identified preferred production strains, evolutionary durations, and conditions for various products. Examples include *E. coli*, *Saccharomyces cerevisiae*, *Corynebacterium glutamicum*, microalgae, and *Streptomyces* sp., which have been applied to elevate the production of metabolites and recombinant proteins (Charusanti et al. 2012; S. Zhou et al. 2003; Otero et al. 2013; Leavitt et al. 2017; L. Y. Jiang et al. 2013; Fu et al. 2013).

It is crucial to note that these new phenotypic traits typically emerge after prolonged adaptive evolution, rather than from short, initial exposures to selection pressures. This method can be scaled up using high‐throughput technologies like liquid handlers, colony pickers, and other automated systems to evolve and screen numerous colonies. With many valuable NPs stemming from the fermentation of actinomycetes, this technique has the potential to explore new avenues for maximizing their yields and could contribute to the identification of novel compounds (Charusanti et al. 2012).

In ALE, microorganisms are subjected to prolonged selection pressure over multiple generations, promoting the accumulation of beneficial mutations. Unlike coculture approaches that primarily rely on interspecies signaling to activate secondary metabolism, ALE focuses on genetic adaptation driven by environmental or competitive pressures. In a study combining ALE with coculture‐based selection pressure, *Streptomyces variabilis* AFP2, sourced from marine environments, was cocultured with *Cryptococcus neoformans* for 30 days, with transfers made periodically. This approach enhanced the anticryptococcal activity of AFP2 compared with the parental strain. HPLC‐UV and GC‐MS were employed to analyze the metabolomics of both the parental and evolved strains. The two strains were assessed for alterations in phenotype and chemotype to identify new traits. The evolved strain produced approximately 21 novel metabolites not found in the parent strain. Among these newly induced compounds were dl‐alanyl‐l‐leucine, dihydro‐3,3‐dimethyl‐2 (3H)‐furanone, enanthamide, 1,3,5‐cycloheptatriene, and 1‐aziridineethanol, which had not been previously reported for antifungal activity or in *S. variabilis*. Additionally, the evolved strain exhibited overexpression of several compounds. Findings from the evolutionary fitness analysis indicated that the evolved strain S3 achieved a 64‐fold (98%) decrease in the growth of *C. neoformans*, with about 52% overexpression of compounds. This study confirms that integrating ALE with coculturing techniques is a valuable strategy for discovering potential molecules with activity against specific pathogens (Rathore et al. 2016).

In a separate investigation involving *S. clavuligerus*, multiple replicates underwent adaptive evolution against methicillin‐resistant *Staphylococcus aureus* N315. This process led to the emergence of a strain capable of constitutively producing holomycin, which was not detectable in the untreated wild‐type strain. In addition, genomic resequencing analysis demonstrated that the evolved strain had lost the sizable 1.8 Mbp plasmid pSCL4 and accumulated several single‐nucleotide polymorphisms in genes that play a role in SM production. This research underscores that competition‐driven ALE can effectively generate mutants capable of either overproducing established antibiotics or potentially uncovering novel compounds (Charusanti et al. 2012).

While classical strain improvement offers a foundational method for generating mutants, ALE provides a more precise and controlled approach, making it particularly valuable in industrial strain development. Classical strain improvement relies on random mutagenesis followed by extensive screening for desirable traits, making it a time‐intensive process with unpredictable outcomes. In contrast, ALE uses directed evolution strategies under defined selection pressures, producing strains with enhanced traits, such as tolerance to harsh environments or increased metabolite yields, over shorter timeframes. However, ALE requires longer experimental durations to observe significant evolutionary changes, whereas classical mutagenesis can produce rapid, albeit less targeted outcomes. Integrating both approaches may provide a robust strategy, combining the broad applicability of classical methods with the precision of ALE.

### 3.7 Coculture Technique

ALE drives strain adaptation to specific conditions, resulting in enhanced resistance, metabolic efficiency, or SM yields. Conversely, coculture techniques create a dynamic environment where microbial interactions can induce the expression of silent BGCs, leading to the discovery of novel metabolites. For example, while ALE is ideal for achieving strain robustness under industrial conditions, coculture techniques excel in uncovering metabolic pathways that remain dormant in monoculture settings. Together, these methodologies provide a holistic approach to strain improvement, addressing both productivity and discovery needs. To maximize the yield of SMs in actinomycetes, replicating their natural habitat through coculturing is essential. Actinomycetes are primarily soil‐dwelling but also establish symbiotic connections. Their mutualistic relationship with living organisms, such as plants, insects, and other organisms, results in the production of SMs that regulate their biological functions or operate as signaling compounds (Ayswaria et al. 2020; Van Moll et al. 2021; Sarmiento‐Vizcaíno et al. 2023; J. Chen et al. 2021). While ALE promotes the accumulation of beneficial mutations over multiple generations, coculture strategies stimulate SM production primarily through ecological interactions between different microorganisms.

Several studies have explored coculturing to activate dormant BGs. Krespach et al. (2023) found that coculturing *Streptomyces iranensis* with *Aspergillus nidulans* led to the production of azalomycin F3a by *Streptomyces*, while *Aspergillus* produced orsellinic acid and other compounds. N. Lee et al. (2020) demonstrated that coculturing *S. coelicolor* A3(2) M145 with *Myxococcus xanthus* triggered the activation of the ACT BGC due to iron competition, significantly increasing ACT production. Onaka et al. (2011) observed that the stimulation of secondary metabolism in *Streptomyces* occurs through cocultivation with mycolic acid‐containing bacteria, resulting in the identification of new compounds, such as catenulobactins and dracolactams.

Additionally, Chevrette et al. (2019) highlighted that *Streptomyces* strains associated with insects demonstrate improved antimicrobial properties, indicating that evolutionary adaptation within the insect microbiome enhances their biosynthetic capabilities. One innovative method for activating SM BGs is to coculture actinomycetes with animal cells. Although the interactions between human cells and intestinal bacteria are well documented (J. Zhang et al. 2021; Nie et al. 2023), the interaction of pathogenic microorganisms with host cells during invasion has also been explored (Toyotome et al. 2020; Hamada et al. 2023). Scientists suggested that pathogenic microorganisms experience stress from immune cells during invasion, which can activate dormant genes and stimulate the production of defensive compounds. In their study, they cocultured pathogenic actinomycetes with J774.1 mouse macrophage‐like cells. This led to the isolation of several compounds in higher yield. Nocarjamide, a cyclic nonapeptide, was first identified in the coculture of *Nocardia tenerifensis* with J774.1 cells. Nocarjamide activated Wnt signaling at concentrations of 20–40 μM, affecting vital processes like tissue formation and cell differentiation (Hara, Arai, Toume, et al. 2018; Cadigan and Liu 2006). Additionally, dehydropropylpantothenamide was also identified using the same coculture method (Hara, Arai, Sakai, et al. 2018).

Despite its success in activating silent biosynthetic pathways and enhancing metabolite production, coculture remains associated with several challenges. Reproducibility can be difficult due to variability in microbial interactions and environmental conditions. Furthermore, scaling coculture systems from laboratory to industrial settings is often challenging because maintaining stable community dynamics can be difficult. Another major limitation is the identification of the producing organism when novel metabolites are detected, particularly when multiple species contribute to metabolite biosynthesis or biotransformation. Advances in metabolomics, isotope labeling, and single‐cell analytical approaches may help address these challenges in future studies.

### 3.8 Challenges and Future Perspective

The optimization of SM production in *Streptomyces* presents several challenges, despite significant advancements in genetic engineering and culture optimization techniques. A major bottleneck is the complexity of regulatory networks governing secondary metabolism, which involves multiple interacting pathways. For instance, the transcriptional regulation of BGCs is often influenced by external and internal signals, making it difficult to predict and manipulate production outcomes. Recent studies have summarized the regulatory cascades of antibiotic production in *Streptomyces*, highlighting the intricate control mechanisms involved (Xia et al. 2020). Moreover, the emergence of antibiotic resistance among microbial populations has increased the demand for novel compounds, pushing the limits of traditional optimization approaches. The genus *Streptomyces* remains a prolific source of new and innovative SMs, underscoring its potential in addressing this demand (Donald et al. 2022).

Another significant challenge is the scalability of laboratory‐based findings to industrial production. Many studies report high yields in small‐scale fermentations, but these often fail to translate effectively to large‐scale fermenters due to challenges in maintaining uniform conditions, such as pH, oxygen transfer, and nutrient availability. Issues in streptomycete fermentation processes for SM production have been documented, emphasizing the need for optimized bioprocess strategies (Barbuto Ferraiuolo et al. 2021). L. T.‐H. Tan (2025) highlights how the inherent phenotypic heterogeneity of *Streptomyces*, including variable gene expression and differentiation states within a population, directly contributes to inconsistent fermentation performance, poor oxygen transfer, and unstable SM yields during scale‐up. It also discusses how single‐cell technologies can help design more robust bioprocess strategies. Furthermore, the metabolic burden imposed on engineered strains can lead to reduced growth rates and instability of the desired phenotype over successive generations, hindering consistent production. The application of regulatory cascades in *Streptomyces* has been explored as a means to enhance antibiotic production, offering potential solutions to these challenges (Xia et al. 2020).

Future research should focus on integrating systems biology and artificial intelligence to overcome these challenges, as computational modeling and machine learning have increasingly been applied to optimize microbial metabolism and fermentation processes (El‐Naggar et al. 2024; Ang et al. 2024; Choi et al. 2019). Systems biology approaches, such as GSMM, can predict the effects of genetic modifications and guide rational strain design. Machine learning algorithms can optimize culture conditions by analyzing vast data sets from fermentation experiments, reducing trial‐and‐error in process development. Recent analyses have provided insights into the modulation of SM production and the regulation of secondary metabolism in *Streptomyces* (Y. Lee, Hwang, et al. 2024). Advances in DNA sequencing technology have significantly surged the number of fully sequenced *Streptomyces* genomes in recent years (N. Lee et al. 2020; Harrison and Studholme 2014). This surge in sequenced genomes has highlighted the importance of bioinformatics tools for annotating and mining their data. A range of tools has been developed for the identification of SMBGCs, such as BAGEL (de Jong et al. 2006), ClustScan (Starcevic et al. 2008), CLUSEAN (Weber et al. 2009), NP.searcher (C. Li et al. 2009), PRISM (Skinnider et al. 2017), and antiSMASH (Blin et al. 2019). Many of these tools leverage conserved sequences within smBGCs to locate them. Findings from genome mining suggest that each *Streptomyces* species possesses approximately 30 SMBGCs, with numerous ones producing yet‐to‐be‐identified compounds, lending credence to the hypothesis that the bioactive capabilities of *Streptomyces* are undervalued (Belknap et al. 2020). Even though genome mining provides a quick way to predict SMBGCs from *Streptomyces* genome data, the characterization of these clusters still involves significant laboratory work, including the activation of dormant SMBGCs, purifying the resultant products, and elucidation of their chemical structures. As such, facilitating the rapid association of products with their corresponding SMBGCs is crucial for enhancing our practical comprehension of SM biosynthetic pathways (N. Lee et al. 2020).

Another promising avenue lies in exploring the untapped biosynthetic potential of silent BGCs. Recent advances in CRISPR‐Cas technologies and synthetic biology offer the tools to activate these clusters and uncover novel bioactive compounds (Y. Lee, Hwang, et al. 2024).

Additionally, coculture techniques, where *Streptomyces* are grown alongside other microorganisms, have shown potential in inducing the expression of silent pathways through interspecies interactions. The submerged cocultivation strategies used for triggering and enhancing SM production in *Streptomyces* have been discussed in recent literature (Boruta 2021).

Finally, the future of SM production in *Streptomyces* lies in the development of sustainable and cost‐effective production systems. The use of agro‐industrial residues as fermentation substrates, coupled with advancements in bioreactor design, can reduce production costs and environmental impact. These innovations, combined with international collaborations and open‐access data sharing, can accelerate progress in the field and ensure the industrial viability of *Streptomyces*‐derived products (Barbuto Ferraiuolo et al. 2021; Donald et al. 2022).

## 4 Conclusion

The pursuit of enhancing the biopotential of *Streptomyces* through strain improvement involves a multifaceted approach integrating classical methodologies with modern techniques. Traditional methods, such as mutation and selection, provide a foundational strategy for generating high‐yielding mutants, although their scope is often limited by the inherent randomness of genetic changes. Genetic engineering offers more precise tools, enabling the amplification or knockout of specific genes, as well as the heterologous expression of biosynthetic pathways from other microorganisms, thereby producing novel metabolites with potentially valuable applications.

Metabolic engineering further refines these approaches by optimizing biochemical pathways and adjusting regulatory elements to maximize the flux of desired compounds. In conjunction with these strategies, ALE enables the selection of strains with enhanced characteristics through prolonged exposure to selective pressures, yielding strains with superior metabolic performance or resistance traits. In‐silico approaches, including modeling and simulation, complement these experimental techniques by providing predictive insights into the effects of genetic modifications, thereby guiding more targeted interventions. Coculture techniques represent a promising frontier in SM production by leveraging microbial interactions to induce novel metabolic profiles that might not be achievable in monoculture. Concurrently, optimizing fermentation conditions remains critical, with adjustments to media composition and process parameters being essential to achieving maximal production yields.

## Author Contributions

**Anum Goraya:** writing – original draft, writing – review and editing, software. **Anila Fariq:** writing – original draft, writing – review and editing, visualization. **Aneela Fatima:** writing – review and editing, writing – original draft. **Syed Hussain Imam Abidi:** writing – review and editing, writing – original draft, validation. **Sohail Ameer Marwat:** conceptualization, software, writing – review and editing. **Gholamreza Abdi:** supervision, writing – review and editing, writing – original draft, conceptualization.

## Funding

The authors have nothing to report.

## Ethics Statement

The authors have nothing to report.

## Conflicts of Interest

The authors declare no Conflicts of Interest.

## Declaration of Generative AI and AI‐Assisted Technology in the Writing Process

During the preparation of this work, the authors used ChatGPT to improve the language and readability. After using this tool, the authors reviewed and edited the content as needed and took full responsibility for the publication's content.

## Data Availability

The authors have nothing to report.
